# The development and validation of a global advanced development framework for the pharmacy workforce: a four-stage multi-methods approach

**DOI:** 10.1007/s11096-023-01585-x

**Published:** 2023-05-14

**Authors:** Sherly Meilianti, Kirsten Galbraith, Lina Bader, Arit Udoh, Desak Ernawati, Ian Bates

**Affiliations:** 1 International Pharmaceutical Federation, Andries Bickerweg 5, 2517 JP The Hague, The Netherlands; 2 Faculty of Pharmacy and Pharmaceutical Sciences, 381 Royal Parade, VIC 3052 Parkville, Australia; 3 Department of Pharmacology and Therapy, Universitas Udayana, Denpasar, 80234 Bali Indonesia; 4 UCL School of Pharmacy, 29-39 Brunswick Square, London, WC1N 1AX UK

**Keywords:** Advancing practice, Competency-based education, Competency framework, International Pharmaceutical Federation (FIP), Pharmacy

## Abstract

**Background:**

Studies have indicated that a generalisable and translatable global framework is a useful tool for supporting career progression and recognising advanced practice.

**Aim:**

To develop and validate a global advanced competency development framework as a tool to advance the pharmacy profession globally.

**Method:**

A four-stage multi-methods approach was adopted. In sequence, this comprised an assessment of initial content and a cultural validation of the advanced level framework. Following this, we conducted a transnational modified Delphi followed by an online survey sampling the global pharmacy leadership community. Finally, a series of case studies was constructed exemplifying the framework implementation.

**Results:**

Initial validation resulted in a modified draft competency framework comprising 34 developmental competencies across six clusters. Each competency has three phases of advancement to support practitioner progression. The modified Delphi stage provided feedback on framework modifications related to cultural issues, including missing competencies and framework comprehensiveness. External engagement and case study stages provided further validity on the framework implementation and dissemination.

**Conclusion:**

The four-staged approach demonstrated transnational validation of a global advanced competency framework as a mapping and development tool for the pharmacy professions. Further study is needed to develop a global glossary of terminologies on advanced and specialist practice. Also, developing an accompanying professional recognition system and education and training programmes to support framework implementation is recommended.

**Supplementary Information:**

The online version contains supplementary material available at 10.1007/s11096-023-01585-x.

## Impact statements

- In this study, a global advanced competency framework for the pharmacy workforce was developed through a four-staged multiple methods approach.
- This framework is a pivotal step in advancing the pharmacy workforce.
- In order to accelerate the advancement of the global workforce in all sectors and settings, transnational collaboration is encouraged.
- An international glossary of terms on advanced and specialist practice is needed.
- As part of framework implementation, it is recommended that a professional recognition system and an education and training programme be developed.

## Introduction

Building a flexible and competent health workforce is the foundation of a strong and sustainable health system [1]. Beyond initial education and training for the regulated health professions, advanced and specialist knowledge and skills are needed to meet and address complex national health, population and individual patient needs. Increasingly, competency-based, time-variable approaches are being used for healthcare education focusing on translating societal health system needs into competencies that must be mastered by the health workforce [2]. Compared to a traditional approach of education where the learning is assumed to be a ‘one size fits all’, the competency based approach is individualised and provides learners with the opportunity to enhance their learning in selected areas once competence has been demonstrated or provide more time if necessary to demonstrate competence. Additionally, the traditional approach focuses on what the trainee knows and the learning experience is defined by fixed curricula and specific time allocations, while a competency-based approach focuses on what the trainee does and the learning experience is guided by the progress of each individual towards competence [3]. The corollary is a demonstration of consistent trustworthiness rather than time spent in training and is facilitated by a robust programme of competency assessment and an accompanying competency development framework [2]. In the health professions, competency development frameworks are now widely used by health students and practitioners to support their learning process and facilitate their development of expertise [4, 5].

The recent decade has witnessed an increasing trend in the use of competency frameworks in the development of the pharmacy profession [6]. These frameworks typically consist of either generic skills for a defined level of practice (such as post-registration foundation or advanced practice) or are sector/role/speciality-specific frameworks [4]. A global survey conducted by the International Pharmaceutical Federation (FIP) in 2015 identified that competency frameworks had been used or were in development in almost 60% of 48 surveyed countries [7, 8]. This survey found global variation in systems to design developmental frameworks for advanced and specialised practice of pharmacy across countries [7, 8]. Adapting frameworks from other countries is seen as a common approach to adopting competency frameworks, reported by 39% of countries with frameworks in place [7, 8]. The impact of national frameworks on advanced practice development and specialisation was also outlined in these studies. In addition, other reports have recognised that the capabilities of ‘advanced’ pharmacists to deliver enhanced patient care and make clinical decisions are at higher levels than those of entry-level pharmacists [9, 10].

The FIP developed the Global Competency Framework (GbCF) in 2012 for practitioners in their foundation level (or early years) practice [11], with an evidence-led update in 2020 [12]. Many countries and organisations have used this framework as a basis to develop their own national foundational framework[13–15], suggesting this set of competencies is globally applicable and valid for foundation practice development. Transnational comparability in existing advanced pharmacy practice frameworks has also been demonstrated [16], highlighting the potential for developing a generalisable and translatable global framework to support career progression and advanced practice recognition. A previously validated framework, Advanced Level Framework (ALF), formerly developed by the UK-based Competency Development and Evaluation Group (CoDEG) [17, 18] has been used as a starting point in developing national advanced competency framework in some countries worldwide. The ALF has been repeatedly tested and validated in different pharmacy specialities in various sectors and levels of practice [19–24] but only within UK professional culture and environments.

### Aim

This paper describes the development and validation of a global advanced competency development framework as a tool to advance the pharmacy profession globally.

### Ethics approval

Ethical approval was not required as the data were neither confidential nor commercially sensitive obtained in this study. However, ethical oversight and support was gained from the International Pharmaceutical Federation (FIP) Executive and Board structures.

## Method

**Fig. 1:**
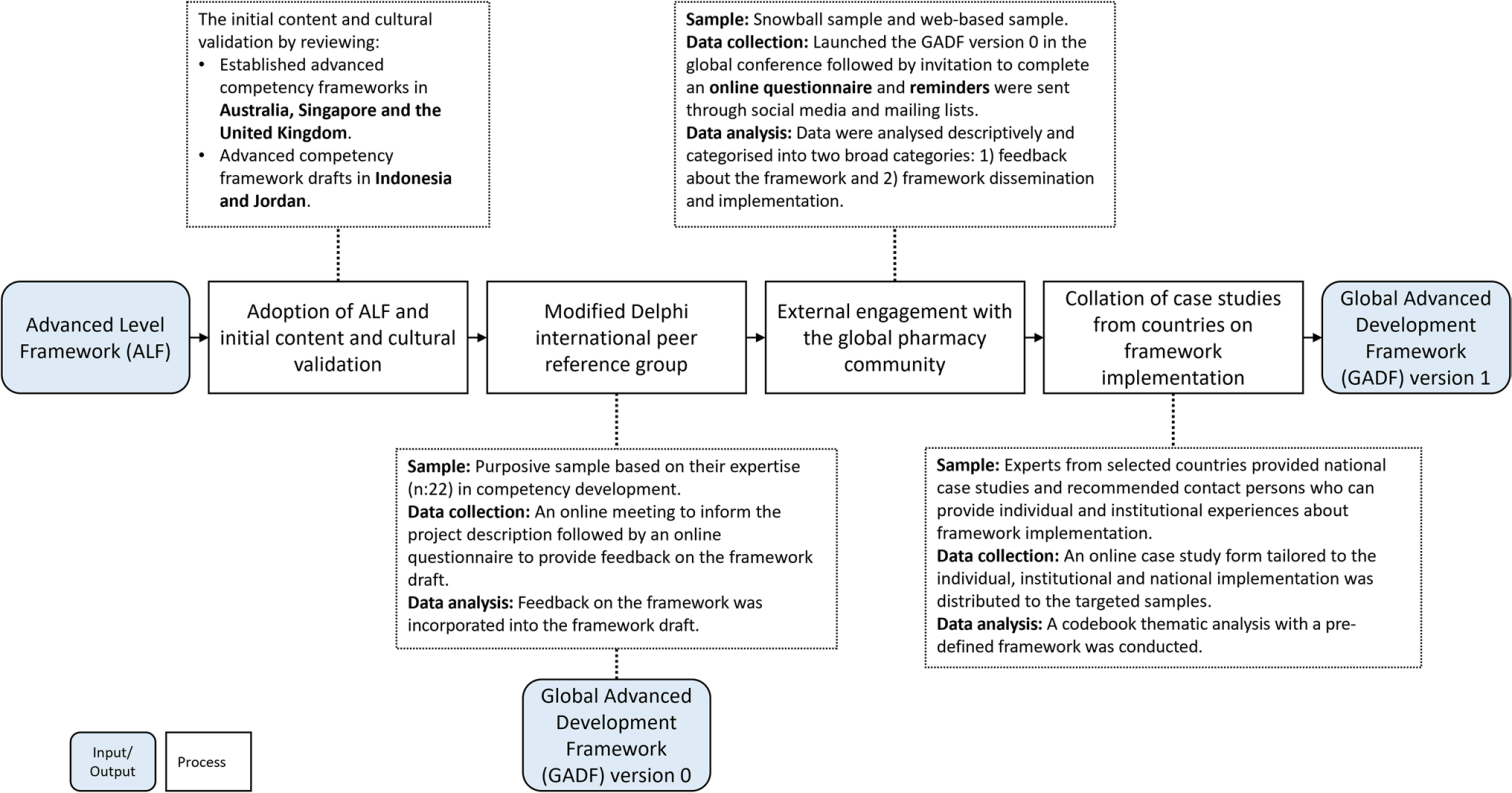
Stages and Stakeholders included in the developement and validation process

This study utilised a multiple methods approach comprising of four stages: (I) assessment of potential adoption of the ALF and an initial content and cultural validation for global workforce; (II) a transnational modified Delphi technique; (III) transnational external engagement with the global pharmacy leadership; and (IV) further validation of the adapted framework using narrative case studies oriented at the individual, institutional and national levels. Figure 1 provides stages and brief methods of the overall development and validation process.

### Stage I: adoption of ALF: initial content and cultural validation

The ALF [17] was the initial reference framework. Content and cultural validation was conducted by reviewing established national advanced competency frameworks in Australia [25], the United Kingdom [26], and Singapore [27], and advanced frameworks under development in Jordan [personal communication] and Indonesia [28]. When this study was conducted, published evidence and personal experience indicated that these were the only countries with national generalised advanced competency development frameworks [4, 7, 8]. The project team, which included representatives from Australia, the United Kingdom, Jordan, and Indonesia, conducted the initial content and cultural validation of the ALF. The validation resulted in a draft Global Advanced Development Framework (GADF) consisting of six clusters with 34 developmental competencies [29]. The first cluster, “Expert professional practice”, is applicable and adaptable for all work sectors and specialities. The remaining five clusters, namely “Building working relationship”; “Leadership”; “Management”; “Education, training and development”; and “Research and evaluation” are generic domains that are applicable independent of the practice sector of pharmacists [29]. Each competency cluster has three defined phases of advancement to support practitioner progression (“Advanced Stage 1”; “Advanced Stage 2”; and “Advanced Stage 3”). “Advanced Stage 1” describes a practitioner who performs well and is at the early stages of advancement. “Advanced Stage 2” describes a practitioner who is an expert in their area of practice, able to manage complex situations and recognised as a leader locally/regionally. “Advanced Stage 3” describes a practitioner who is recognised as a leader in an area of expertise (nationally, and often internationally) [29].

### Stage II: transnational modified Delphi

This draft framework was further validated using a transnational one round modified Delphi peer reference group. It was conducted between 21st August and 2nd September 2019. In contrast to the usual Delphi technique [30], no ratings were used in the feedback process, and the agreement method was implicit (qualitative) rather than explicit. Twenty-two expert members from 16 countries (see Table 1) were purposively recruited from the FIP global expert network based on their expertise in competency development processes in their organisation. The experts were initially briefed in an online meeting about the project’s aims. They were subsequently invited to provide feedback on the framework using an online questionnaire. The online questionnaire was developed by the project team, in which the questionnaire items elicited feedback on any gaps or missing competencies; any cultural, language and conceptual issues with competencies; comprehensiveness of the framework’s three stages of advancement (i.e. whether they were reasonable and understandable); and general comments (see Supplementary material 1 for the lists of questions). The questionnaire was completed by twenty-one experts, with one expert not responding. The feedback was collated thematically into a Microsoft Excel worksheet. Subsequently, a collaborative online meeting was involving the project team members to discuss and integrate the feedback into the framework. Following this, the draft framework (GADF version 0) was created and launched for broader profession-wide engagement and feedback at a global conference in September 2019 [31].

**Table 1:** Study’s stage and sample description

Stage	Sample description
1. Initial content and cultural validation (n: five countries).	Two categories of countries: a. Countries with established advanced competency frameworks: Australia, Singapore and United Kingdom b. Countries with advanced competency framework drafts: Indonesia and Jordan
2. Transnational modified Delphi peer reference group (n: 21 experts).	Twenty-one experts with the following countries and expertise: a. *Country* Algeria (1), Argentina (1), Australia (2), Canada (1), Croatia (1), Indonesia (1), Japan (1), Jordan (1), Nigeria (2), Portugal (1), Qatar (1), Sudan (1), Syria (1), Netherlands (3), United Kingdom (1), and United States (2) b. Expertise in* The competency development process (4), Education and training (6), Interprofessional collaboration (1), Leadership (8), Management (17), Policy and advocacy (2), Pharmacy practice and service development (4), Research and evaluation (13), and Scientific development (3)
3. Transnational external engagement with the global pharmacy leadership community (n: 29 experts).	Twenty-nine responses were received from the following countries and broad affiliations: a. *Country* Algeria, Germany, Ghana (3), India, Indonesia, Kuwait, Malawi, New Zealand, Nigeria (2), Norway, Pakistan (3), Portugal, Rwanda, South Africa, Switzerland, United Arab Emirates, Zambia, Zimbabwe (2), and do not provide country information (5) b. *Expertise* Worked in university (n:10), practitioners (n:7), professional organisation (n:5), and affiliation not provided (n: 7)
4. Case studies from countries at the individual (n: 7), institutional (n:2) and national (n: 5) levels.	a. Seven individual case studies from: Australia (a hospital pharmacist); Singapore (3 hospital pharmacists and a primary care pharmacist), and United Kingdom (2 hospital pharmacists) b. Two institutional case studies from Australia and United Kingdom (university setting) c. Five national case studies (Australia, Singapore, United Kingdom, Indonesia and Jordan)

*One expert can have multiple expertise

### Stage III: transnational external engagement with the global pharmacy leadership community

Simultaneously with the launch of the framework draft, an online questionnaire was distributed to the global pharmacy community to seek feedback on continuing relevance and validity of the framework. The global community refers to FIP member organisations, FIP members and any pharmacists who access the online questionnaire through their network. The questionnaire was adapted from the outcome of the modified Delphi technique stage (see Supplementary material 1). The survey was open for six months, and two reminders were sent through the social media network of FIP and the mailing list of FIP members. Feedback obtained from the survey was analysed in textual form and categorised according to the following themes: (1) feedback about the framework, (2) its dissemination, and (3)implementation. The initial categorisation which was conducted by the main author was further reviewed by the other researchers for consistency and validity.

### Stage IV: collation of case studies from countries on framework implementation at the individual, institutional and national levels

To gain further insights on framework implementation, an invitation was distributed to the experts in countries with demonstrated expertise in the adoption of an advanced competency framework. The countries included those with existing national advanced competency frameworks and these were Australia, Singapore, the United Kingdom, Indonesia and Jordan. Experts who were involved in the development and implementation of the national advanced competency frameworks in the respective countries were invited to share their experiences. The experts were also asked to suggest contact persons who could share implementation experience at the institutional level and personal experience using the framework. An online case study form tailored to the individual, institutional and national implementation was developed (see Supplementary material 1 for the question list). A codebook thematic analysis with a pre-defined framework building from case study questions was carried out [32]. For institutional and national case studies, the pre-defined framework was (1) Drivers, (2) Development process, (3) Implementation, and (4) Impact. While for the individual case study, the pre-defined framework used was (1) Context of using the framework; (2) Use of the framework for professional and/or career development; (3) Additional tools and resources which could assist engagement with the framework; and (4) Barriers to utilising the framework. The themes were defined as topic summaries of responses to the case study questions.

## Results

### Sample description

Table 1 provides information on the sample description for each stage. The five countries included in the initial validation and case studies gathering were: Australia, Singapore, the United Kingdom, Indonesia and Jordan. Experts from 16 countries participated in the modified Delphi stage and experts from additional 15 countries provided feedback during the external engagement stage. In total, 31 countries were represented in this study.

### Feedback about the framework

Modifications to the ALF were mostly made during the transnational modified Delphi peer reference group (stage II). Most modification was to adapt the competency items and descriptors to be applicable across sectors and scopes of practice of pharmacists. In terms of the comprehensiveness of the competency staging, some experts queried whether the framework applies beyond a few years of pharmacists’ career (foundational stage) or if it applies from day 1 of their professional career. Some feedback received was also related to the need for more explanations to describe some terms that may differ across countries, such as core and defined areas, defined practice, resources (scope of resources to be managed), etc. Table 2 provides a summary of suggestions to the framework.

**Table 2:** Modifications to the framework

Part of the framework	Suggestions/modifications	Actions to the framework and relevant competency items
*Cluster 1. Expert professional practice*		
Expert skills and knowledge	A missing gap related to prescribing practice	This feedback was not incorporated considering prescribing practice may not be available in all countries
Patient care responsibilities	This competency may not be relevant to non-patient-facing role (e.g., academic, industry, regulatory, pharmaceutical sciences)	This competency: “patient care responsibilities” was modified to “developing professional expertise”
Reasoning and judgement	It might be good to have some wording related to problem solving	“Problem-solving skills” was added to this competency item
*Cluster 2. Working with others*		
Communication	“Patients, colleagues and clinicians” seems to be only relevant for those working in patient care setting Consider adding a component of empowerment in communication skills Perhaps there is need to have a separate competency for “communication” with patients and other healthcare professionals	“Patients, colleagues and clinicians” was modified to “relevant stakeholders” An ability to “empower” was added to this competency item This feedback was not incorporated into the framework to provide a broader and flexible concept of global workforce advancement
Teamwork and consultation	“Across boundaries” needs to be described further	“Across boundaries” was modified to “across boundaries (profession/sector/area)”
*Cluster 3. Leadership*		
Strategic context	The descriptor of advanced stage 3 at the “strategic context” competency item, which is to “create national healthcare policies”, might not apply to practitioners	This descriptor was modified to “demonstrate active participation in creating relevant local, national, regional or global policies.” This is because the focus of this competency item was about active participation in the policy creation, which could be done at the local, national, regional or global levels.
Governance	“Clinical governance” was deemed challenging to translate in other languages.	“Clinical governance” was modified to “Standard, quality and accountability”
Innovation	It is not clear what “requires limited supervision” means	“Required limited supervision” was modified to “often requires supervision for others”
*Cluster 4. Management*		
Managing change	The descriptors for advanced stage 3 of “managing change” should include the ability to promote, initiate and/or lead a process of change, not only to manage change	This descriptor was modified to “Demonstrates ability to promote, initiate and/or lead a process of change at a higher level”
Strategic planning	The experts suggested further descriptions of the competency descriptor related to the “strategic planning” by considering countries’ political and economic instability	This descriptor was modified to include skills in “adapting the planning based on organisational politics changes in the internal and external environment”
*Cluster 5. Education, training and development*		
Conducting education and training	This competency is not only about conducting teaching effectively but also to deliver teaching and feedback effectively Perhaps this competency could refer to advanced degrees or credentials in the education part.	Conduct teaching efficiently was modified to “Deliver teaching and feedback effectively” This feedback was not incorporated into the framework to provide a broader and flexible concept of global workforce advancement
Links practice and education	Involvement in the education and training in some countries is not only related to formal education but also in other ways	The stage 1 descriptor, which includes “formal education” was modified to “Participates in the delivery of education and training”
Educational policy	Policy on workforce does not only include education, but also planning and development	“Workforce education” was modified to “workforce education, planning and development”
*Cluster 6. Research and evaluation*		
Develops and evaluates research protocols	There was no research structure in the clinical setting of this competency—so this competency may not be relevant in the country	This feedback was not incorporated into the framework because this competency was essential for advancing pharmacy practice
Establishes research partnerships	The word “Specialist” in the competency descriptor provides a narrow context of competency	Remove “specialist” in the “specialist research” to provide a broader term to the description
*Any other comments*		
Stages in the framework -Advanced stage 1 -Advanced stage 2 -Advanced stage 3	The application of the staging related to if this framework applies beyond a few years of pharmacists’ career (foundational stage) or if it applies from day 1 of their professional career. Also, how this framework relates to the current global competency framework for early career pharmacists	The staging levels are further described in the footnotes

### Feedback about framework dissemination and implementation

Respondents in the modified Delphi (stage II) suggested development of a handbook to guide the use of the framework. In addition, a glossary defining some of the main concepts contained in the advanced competency framework was deemed necessary, considering the concept of advancement was new in some countries.

*“Clinical governance is a term that widely western countries (or more focusing on UK system). I do not think non-English speaking countries would be able to understand this.” [Japan]*.

Some respondents commented on the staging within the framework and the means to assess this. They queried how the staging relates to years of experience, position in the workplace, and the use of scoring metrics for the assessment. This was further explained in the handbook [29], in which the emphasis is on the development of competencies from the Global Competency Framework (foundation level) towards the advanced framework stages 1, 2 and 3, and not on the examination of practitioners.

*“Will the GADF be associated with recommendations for the development of formal career progression systems at country level, that are linked to competency assessment and certification (and that could eventually be linked to requirements for certain positions, or with remuneration scales, for example)?” [Netherlands]*.

Feedback obtained during external engagement with the global pharmacy community (stage III) recommended a stakeholder engagement to support the dissemination of this framework including local and national pharmacy organisations, universities, students, and early career organisations. Another suggestion was to translate the concept into global and local statements and then into national actions and priorities.

*“The basic concept should be translated into actual and up-to-date adapted global and local statements which from the strategic perspective translate into (national) actions and priorities with specific topics depending on regulatory and legal conditions for pharmacists working in the different pharmaceutical areas.” [Switzerland]*.

To support the implementation of the framework at the national level, it was suggested that an online platform consisting of tools and instructions on how to use the framework should be developed to accompany the framework. This platform should facilitate personalised results and frequent self-evaluation reminders, informing which areas and clusters practitioners are good at and need to develop further.

*“I think it would be helpful to use this as a Member Organization (MO) benefit. I suggest that FIP develops some guidance documentation around how this Framework can be used.” [New Zealand]*.

On a global level, building a database of training mapped to this validated GADF was suggested, to share examples for individual nations and regions to use in their advancement towards global desired standards.

*“Let us build the Global framework of Global Pharmacy Training development and practice embracing individual national and regional towards global desired standards.” [Zambia]*.

Working collaboratively with other healthcare professionals was also recommended to test this framework and it was also suggested the need to conduct cost-benefit evaluation to demonstrate how the framework implementation impacts patients.

*“Advertising and marketing this to physicians and other healthcare professions. Cost-benefit evaluation to demonstrate all of this. [At the end] it’s the patient.” [United States]*.

### Framework drivers, development, implementation and impact at the national and institutional levels

At the national level, one of the drivers of advanced competency framework development reported in the United Kingdom was the need for pharmacy education and training transformation to support pharmacists’ role in patient safety. Ongoing development in several designated areas of speciality practice reported in Australia triggered the need to look at what would be a useful curriculum, roadmap and developmental framework. Another driver reported in Singapore was a need for a clear career pathway to support and motivate pharmacists throughout their careers. The career pathway could be supported by a framework that defines expected competency levels for pharmacists at different levels of seniority and experience. These drivers were also similar to the reason reported in the institutional case studies on why they adopted the competency framework in their organisation, namely, to support the education and development of their staff, to formalise advanced pharmaceutical care skills and competencies recognition, and to have clear and identified pathways for further practitioner advancement following on from initial post-registration foundation training.

There were some variations in competency framework development reported in the national case studies. In Australia for example, the development process included groups of all pharmacy bodies who agreed on national core competencies, which were then adopted by member organisations in the country. A similar approach was seen in the United Kingdom, where the professional leadership body established an expert group to review the framework and reflect practice across all sectors and scope of practice. The Singapore case study described a collaboration with the Ministry of Health in initially developing the framework, followed by the introduction of the framework in healthcare institutions. In Indonesia and Jordan case studies, the development included translation, adoption and adaptation, and national engagement phases to ensure the framework is culturally applicable. Institutional case studies reported that the development process included referring to the curriculum or training programme to map with the competency framework.

Across the case studies, framework implementation at the national level was sometimes accompanied by a recognition or credentialing system to award advanced practitioner status. For instance, in Australia, a portfolio-based impact demonstration was utilised with an advisory group steering the process. The framework was then further incorporated into the national competency standards to outline a clear journey of increasing performance for each domain and competency. In Singapore, the framework was implemented as a developmental tool, and a guidebook was developed so practitioners could use the framework actively in practice. Portfolio training workshops and regular engagement with pharmacy leaders were also conducted to promote the adoption of the framework. In the United Kingdom, the framework is used for the consultant credentialing pathway. Institutional case studies reported that the advanced competency framework was implemented as part of a suite of training programmes and as a basis of a portfolio where students collected workplace evidence mapped to the framework.

Some impacts of the framework implementation were highlighted in the submitted case studies. The case study of Singapore reported that the advanced framework has allowed senior pharmacists to advance systematically since it was first introduced in 2016. In the United Kingdom, the advanced framework has underpinned a formal credentialing process for consultant pharmacists which has been recognised across professions. In Australia, in alignment with the national foundation competency framework, the advanced framework provides a clear career path for pharmacists from when they were students. Also, a formal recognition system of pharmacists’ stages of advancement was developed, and linked to remuneration [33].

### Utilisation, support needed and barriers in using the framework from individual perspectives

Table 3 provides information on how individuals use the framework, what additional tools and resources are needed to support the framework implementation, and what barriers exist to the framework implementation. From the context of using the framework, most individual case studies highlighted the use of the framework for their personal growth and development. They also used the framework to map toward national standards and link with their scope of practice and job roles. Practitioners used the framework for professional and career development to develop their portfolios, support their job applications, and support other team members in building their portfolios. Individual case studies also described the use of the framework in the workplace, related to providing evidence of advancement, attaining specific job grades linked with promotion, and developing targeted programmes for staff in the workplace. Case studies informed a need for tools and resources to support the framework, workshops or courses related to framework implementation, support in mentoring and coaching, and others related to engagement with stakeholders and recognition in the job roles. Case studies highlighted personal barriers in the framework implementation, particularly related to time-consuming processes and value for pharmacists. Incentives and motivations were deemed beneficial for the successful implementation of the framework.

**Table 3:** Utilisation, support needed and barriers in using the framework from individual perspectives

Category	Findings
Context of using the framework	**For personal growth and development** 1. To track self-growth, progress and achievement over time; 2. To facilitate self-reflection in order to review where they are and to acquire skillsets for self-directed learning; 3. To focus on targeted areas for personal growth and development. **To map towards national standards and demonstrate impact for patient care** 1. To work towards national pharmacy standards; 2. To collate evidence for detailing career and impact on patient care. **To link with scopes of practice and roles** 1. To benchmark in taking specific ‘advanced’ practitioner roles; 2. To chart journey based on job scope.
Use of the framework for professional and/or career development	1. **To develop a personal portfolio**: Portfolio guides career development, track progress, and identifies areas for improvement. 2. **To support the job application**: By supporting the application to attain a consultant post. 3. **To support other team members**: Mentoring and guiding other pharmacists through the portfolio development process and mentoring them to identify their strengths and weaknesses for addressing their developmental gaps. 4. **To provide evidence of advancement in the workplace**: As a result of pharmacists being recognised as advanced practitioners, employers will have tangible evidence of their employees’ enhanced capability. 5. **To attain specific job grades –** linked with promotion: This framework has been mapped to the job grades. Each job grade must achieve a specific competency level in a specific area. 6. **To develop targeted programmes for staff in the workplace**: With the framework in place, workplace managers can identify common weaknesses among their employees and devise training programmes to address them.
Additional tools and resources which could assist engagement with the framework	**Tools and templates to support the framework** 1. Provide templates and examples of portfolios for practitioners on similar career paths. 2. Provide an online modifiable portfolio template where practitioners can build their portfolios and upload evidence in the system. 3. Provide a training roadmap to help practitioners identify the courses required to reach their desired performance level. **Organise workshops or courses related to**: 1. Reflective writing; 2. Portfolio building and assessment; 3. How to gather effective evidence; 4. Peer review learning; 5. Providing feedback. **Provide support in mentoring and coaching** Provide support to set up mentors or coaches who can provide feedback and facilitate the development of practitioners; this could be organised in the workplace. **Other supports** 1. Provide resources to allow and support pharmacists to protect their time in reflecting and writing up their portfolios. 2. Organise peer review sessions. 3. Engage with other professional organisations to foster collaboration in providing an impact on patient care. 4. Engage with the early career workforce to introduce them to the idea of an advanced practice framework at an early stage and help them incorporate it into their long-term career goals. 5. Promote the recognition and utilisation of the framework in the job roles. 6. Promote the use of the framework as part of re-validation processes for pharmacists.
Barriers to utilising the framework	**Micro-level barriers - individual** 1. The process of collecting evidence and building a portfolio is time-consuming and intensive. There is no time allotted for personal growth and advancement in individual job plans. 2. Lack of pharmacist’s motivation, where pharmacists may not see the immediate benefits of creating their portfolio as a value-add to their career development. 3. Questions on the value of this framework for senior practitioners remain related to what happens when a practitioner attains expert for all domains. 4. The unfamiliarity of the process of gathering evidence for portfolio and understanding the competency standards. 5. The ability to write reflectively varies greatly between individuals; this may affect practitioners’ integrity and morale. **Meso-level barriers – workplace** 1. A flat organisational chart and lean staffing structure create difficulty for practitioners in collating evidence for advancement. 2. Limited link on how professional recognition system or credentialling provide additional benefits in the workforce or additional roles, responsibilities, or remuneration. 3. There are a limited number of vacancies for promotion even if practitioners have the necessary skills and experience to move up in the organisation. **Macro-level barriers -national** 1. How to get buy-in from the whole workforce since the framework is not ‘mandated’; 2. How to implement the framework consistently throughout all the institutions within the nation; 3. Lack of specific performance indicators for assessing the framework’s effectiveness and impact; 4. Lack of education supervisors to support in mentoring and coaching.

## Discussion

### Summary of findings

When this study was conducted, there was no global advanced pharmacy framework, which highlighted an opportunity to develop one to support countries in advancing their pharmacy workforce. This paper describes the development and validation of a global advanced competency framework for pharmacy and adds further evidence on developing a global framework from an established, previously validated framework [18, 24, 28, 34]. The focus of the development and validity is on the generalisability and applicability of the framework within its original cultural context.

### Implications for policy, practice and research

Stage I of the framework development included initial country adoption of this framework draft in non-anglophone countries (i.e. Jordan and Indonesia), providing evidence of cultural acceptability and applicability of the concepts of advancement within the framework. Country adoption experience indicated that some anglophone concepts attributed to ‘advanced practice’ do not translate easily. For example, a recent study in Indonesia identified some terminologies that were not common in Indonesian cultures [28]. Healthcare semantics are important considerations in advanced practice competencies, highlighting the importance of having agreed terminologies on competencies. Further work is being undertaken to develop a global glossary to describe terminologies within advancement and specialist practice. Having a global glossary shared across countries could support the translatable policy and tools and facilitate collaboration between countries [4, 35].

Stage II of framework development utilised a modified Delphi technique. From five characteristics of classical Delphi techniques identified in the literature [30, 36], this study utilised a standardised questionnaire, private decisions elicited, feedback shared with the group and an opportunity for the experts to provide further feedback. The modification of Delphi lies in the aggregation methods whereas, in classical Delphi, statistical analysis was used. In this study, a qualitative analysis was utilised to focus on the modification to be made to the framework. Expert feedback suggested some modifications to the competency phrasing to add greater clarity to some components of the framework.

Stage III of framework development (transnational external engagement with the global pharmacy leadership community) offered additional validation data for this framework and ensured that the framework fits the demands of the global pharmacy workforce as a mapping and development tool. The strength of this paper is the involvement of a diverse scope of national leadership organisations and individual practitioners in the development process.

Stage IV gathered case studies and insights derived from examples of how the framework has been implemented in the individual, institutional and country contexts [29]. Case studies provided further validation of the use of the framework in developing pharmacists’ competency and facilitating performance across practice sectors [24, 37–39]. This framework could have interest and applicability for professional leaders, national organisations, educators, regulators, and practitioners to work towards the global advancement of pharmacy practice. A collaborative approach across organisations in the country is encouraged to support the implementation of the framework. Partnership and collaboration between national and global organisations have also been shown effective in accelerating the process of competency framework development in countries [9, 28, 35]. Having the global advanced competency framework could provide the global pharmacy community with a starting point for developing their own framework. This advanced framework also provides a tool to facilitate competency-based approaches to education and training, policy, regulation, pharmaceutical care services and the health workforce, which will support the alignment of the competency towards health needs and high-quality care delivery.

This advanced competency framework opens opportunities to develop an accompanying professional recognition system to support the framework implementation. It also opens opportunities to develop a national training programme (NTP) built on the framework, such as the ongoing programmes organised in Indonesia [28] and Singapore [34]. An advanced clinical skills course coupled with a training program may facilitate the transition of pharmacists to autonomous advanced pharmacy practice [40]. A series of train-the-trainer (TtT) programmes to prepare and train local trainers to build their capacity to use the framework has been organised in Indonesia [41]. The use of the advanced competency framework and a robust training and assessment programme will upskill the national pharmacy workforce more rapidly and in a sustainable way [35].

### Strength and limitations

The strengths of this paper are its approach to the entire workforce, its description of a framework which was developed by adopting and adapting an existing framework which has its validity in its original context development, the collection of evidence from diverse viewpoints, and its focus on the relevancy and applicability across a broad range of career options for pharmacy. However, the limitation of this is that specific information could not be included because the core competencies are broad and generic. Also, it needs to be noted that feedback from the experts could have been influenced by each individual’s area of practice rather than representing global or national viewpoints. Despite best efforts, some regions, and pharmaceutical scientists, are still underrepresented among the participants of modified Delphi and the global community engagement. During the public consultation, due to limited resources, the questionnaire was only available in English which may prohibit potential non-English speaking participants from engaging. However, since the framework has been launched several translations have been provided to enable further monitoring and evaluation of the framework [31]. Obtaining views from other stakeholders such as patients, other non-government and government organisations, employers, health care providers and other professions may provide further validity to the framework implementation.

## Conclusion

This paper describes the development and validation process of a global advanced competency framework with the basis of applicability and implementation of the framework at the individual, institutional and national levels. This framework offers opportunities for transnational collaboration and could be used by practitioners, institutions and policymakers as a template to develop their own framework to support professional development and recognition of its pharmacy workforce. Further study to develop a glossary of terminologies is needed to facilitate ongoing collaboration. In addition, developing an accompanying professional recognition system and education and training programmes to support framework implementation is recommended. This framework is a pivotal step to advancing the pharmacy workforce globally.

## Electronic supplementary material

Below is the link to the electronic supplementary material.

Supplementary Material 1
