# Supplementary material for: The development and validation of a global advanced development framework for the pharmacy workforce: a four-stage multi-methods approach
Source: Int J Clin Pharm. 2023 May 14;45(4):940–51. doi: 10.1007/s11096-023-01585-x (PMC10366019; doi:10.1007/s11096-023-01585-x)
Supplement: Supplementary file 1 — Supplementary Material 1 [file 11096_2023_1585_MOESM1_ESM.docx]

Supplementary material 1: Question list used in stages II, III and IV of framework validation

| **Stage** | **Question** |
| --- | --- |
| **Stage II: Transnational modified delphi peer reference group** | 1. Are there gaps in the competencies or areas of general (and generic) practice advancement that are omitted?    1. Yes, please provide more explanation on which competencies or clusters that you think are missing from this framework.    2. No. 2. Are the current 3 stages of competency development/progression (within each cluster) reasonable and understandable over the course of a nominal career progression period?    1. Yes.    2. No, please provide some commentary. 3. Are there any cultural, language or conceptual issues with the competencies or clusters as they are currently written?    1. Yes, please provide ideas and suggestions for overcoming the issue.    2. No. 4. Please provide some general commentary for us, for example, “This is a good thing…” or “I can see some issues with…that could be overcome by…” or “We now need dissemination or communication implementation plans…” Something that would be helpful to the Workforce Development Goal 4 (WDG4) Team and FIP. |
| **Stage III: Transnational external engagement with global pharmacy leadership community** | - 1. Are there obvious gaps in the high-level clusters and competencies?      1. Yes, please provide more explanation on which competencies or clusters that you think are missing from this framework.      2. No.   2. Are the current 3 stages of competency development/progression (within each cluster) reasonable and understandable over the course of a nominal career progression period?      1. Yes.      2. No, please provide some commentary.   3. Are there any cultural, language or conceptual issues with the competencies or clusters as they are currently written?      1. Yes, please provide ideas and suggestions for overcoming the issue.      2. No.   4. Please provide some general commentary, for example on the dissemination or communication implementation plans for this Framework that would be helpful to the project team and FIP.   5. Are you interested in supporting or initiating the implementation of the GADF in your member organisation? |
| **Stage IV: Collation of case studies from countries on framework implementation at the individual, institutional and national levels** | **Country experience on adoption and adaptation of ALF**   - 1. How did your country decide to adopt and adapt the CODEG ALF?   2. Please describe the process of how your country adopted/adapted the CoDEG ALF.   3. How has the adopted/adapted framework been implemented?   4. What are your country's future plans for use of the framework?   5. Could you describe the impact of the framework implementation in your country (e.g., remuneration of pharmacists, recognition by government and other health care professionals)?   **Institutional experience on adoption and adaptation of ALF**   1. How did your institution decide to adopt and adapt the CODEG ALF? 2. Please describe the process of how your institution adopted/adapted the CoDEG ALF. 3. How has the adopted/adapted framework been implemented? 4. Please provide a copy of the framework (attached file or link). 5. What are your institutions’ future plans for the use of the framework? 6. Please provide any publications that describe this work (attached files or links). 7. Any other comments.   **Individual experience using an advancing practice framework**   1. How did you find out about the framework? 2. In what context do you use the framework (e.g. career development, self-reflection, etc.)? 3. What additional tools and resources would assist your engagement with the framework (e.g., CPD offerings, courses, workshops, etc.)? 4. How do/could you use the framework for professional and/or career development? 5. What are the barriers to utilising the framework? 6. Any other comments. |
